# Revealing the Low-Temperature Phase of FAPbI_3_ Using a Machine-Learned Potential

**DOI:** 10.1021/jacs.5c05265

**Published:** 2025-08-14

**Authors:** Sangita Dutta, Erik Fransson, Tobias Hainer, Benjamin M. Gallant, Dominik J. Kubicki, Paul Erhart, Julia Wiktor

**Affiliations:** † Department of Physics, Chalmers University of Technology, SE-41296 Gothenburg, Sweden; ‡ School of Chemistry, 11248 University of Birmingham, Edgbaston B15 2TT, United Kingdom

## Abstract

Formamidinium lead iodide (FAPbI_3_) is a material of interest for its potential in solar cell applications, driven by its remarkable optoelectronic properties. However, the low-temperature phase of FAPbI_3_ remains poorly understood, with open questions surrounding its crystal structure, octahedral tilting, and arrangement of formamidinium (FA) cations. Using our trained machine-learned potential in combination with large-scale molecular dynamics (MD) simulations, we provide a detailed investigation of this phase, uncovering its structural characteristics and dynamical behavior. Our analysis reveals the octahedral tilt pattern and sheds light on the rotational dynamics of FA cations in the low-temperature phase. Strikingly, we find that the FA cations become frozen in a metastable configuration, unable to reach the thermodynamic ground state. By comparing our simulated results with experimental nuclear magnetic resonance (NMR) and inelastic neutron scattering (INS) spectra, we demonstrate good agreement, further validating our findings. This phenomenon mirrors experimental observations and offers a compelling explanation for the experimental challenges in accessing the true ground state. These findings provide critical insights into the fundamental physics of FAPbI_3_ and its low-temperature behavior, advancing our understanding of this important material.

## Graphical abstract

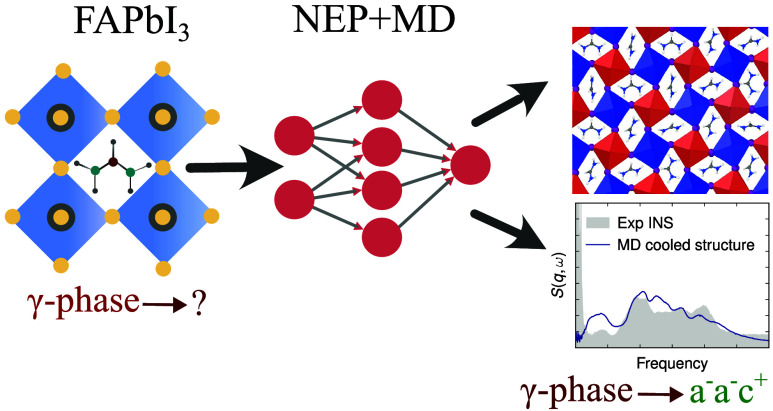

## Introduction

Perovskite solar cells are recognized as promising optoelectronic devices due to their band gap favorably matching the solar spectrum.^1–5^ Among various materials, hybrid halide perovskites, particularly methylammonium lead iodide (MAPbI_3_) and formamidinium lead iodide (FAPbI_3_), have attracted significant attention for next-generation photovoltaics. Their efficiency has rapidly increased beyond 25% since their initial application.^2,5–7^ However, stability issues remain a major limitation, driving research into their crystal structure dynamics and phase bahavior.^8–13^ Previous studies have highlighted the crucial role of rotational dynamics of organic cations and octahedral tilting in hybrid halide perovskites, influencing not only phase stability but also carrier lifetimes and overall device performance. Neglecting these dynamics can lead to misinterpretations in experimental studies, particularly for techniques sensitive to local structural variations.^14–18^

FAPbI_3_ has emerged as a preferred choice for photovoltaic thin films due to its superior optoelectronic properties.^7^ It is found to be cubic (*Pm*3̅*m*) at 300 K. After cooling to below 285 K, the material was found to undergo a phase change to a tetragonal β-phase (*P*4/*mbm*). Another phase transition was noted at 140 K to a phase designated γ.^19–23^ Notably, ambiguity persists regarding the nature of the low-temperature γ-phase, with several experimental studies suggesting possible structural disorder.^5,7,12,15,20,22,24,25^ Fabini et al. observed a substantial blue shift in photoluminescence spectra during the β to γ phase transition in FAPbI_3_, attributed to reduced Pb–I orbital overlap due to symmetry lowering. Particularly, the blue shift persists despite reduced octahedral tilting, suggesting crystallographically hidden disorder in the Pb–I network without disorder-induced emission broadening.^19^ Weber et al. reported that while FA cations exhibit restricted orientations in the mid-temperature tetragonal phase, they display high levels of disorder below 140 K in a glassy state, with residual long-range ordering and octahedral tilting contributing to additional diffraction intensity.^20^ However, the exact nature of this disorder remains unresolved. In this work, given the need for a detailed understanding of the low-temperature crystal structure and FA dynamics, we employ atomic-scale simulations to investigate the microscopic behavior of the γ-phase.

Computational studies of halide perovskite structures face challenges due to the strong anharmonicity of these materials and the rotational degrees of freedom of the organic cations. Conventional static calculations provide limited insight, while perturbative approaches are hindered by the strong anharmonicity, necessitating molecular dynamics simulations to capture finite-temperature effects. However, ab initio MD simulations are computationally expensive, restricting access to long time scales and large system sizes. Recently, machine-learned interatomic potentials have emerged as powerful tools for studying halide perovskite dynamics, enabling efficient sampling without compromising accuracy.^8,26–31^

Here, we employ a machine-learned interatomic potential recently developed for the MA_1–*x*_FA_*x*_PbI_3_ system,^32^ based on the fourth-generation neuroevolution potential (NEP) framework,^33,34^ to analyze the atomic-scale dynamics of FAPbI_3_ via MD simulations. Notably, the machine-learned potential accurately reproduces all known phases of FAPbI_3_ reported in the literature.^5,7,12,20,22–25^ We first identify the ground-state structure as *a* ^–^ *b* ^–^ *b* ^–^ (or, *a* ^0^ *b* ^–^ *b* ^–^) in Glazer notation.^35^ We then analyze the octahedral tilting and FA molecular orientation across different phases. Our simulations reveal that the low-temperature phase exhibits an *a* ^–^ *a* ^–^ *c* ^+^ structure due to kinetic trapping in a metastable state during cooling. To understand this phenomenon, we further investigated the complex dynamics of organic cations, their correlations, and the associated free energy landscape.

## Methods

### MD Simulations

MD simulations were carried out using the gpumd package with a time step of 0.5 fs. We use a NEP trained potential for a mixed FA_1–*x*_MA_*x*_PbI_3_ system as described in ref 32. The potential was trained against density functional theory (DFT) data generated using the SCAN+rVV10 functional.^36^ The reference data comprised a wide range of configurations representing FAPbI_3_, MAPbI_3_, and mixtures thereof. The model, as well as the training data, are available on Zenodo (10.5281/zenodo.14992798). We employed the Bussi–Donadio–Parrinello thermostat^37^ and the stochastic cell rescaling (SCR) barostat^38^ method to control the temperature and pressure, respectively. A system of 49152 atoms was chosen to avoid finite-size effects^39^ (see Figure S4). We ran heating and cooling MD simulations in the NPT ensemble within a 0 to 350 K temperature span with different heating and cooling rates. Further details on the MD analyses, including structural and dynamical characterizations, are presented in the Results section.

### NMR Measurements

In order to determine the local environment of FA in the γ-phase of FAPbI_3_, we carried out low-temperature magic angle spinning (MAS) solid-state ^13^C and ^15^N nuclear magnetic resonance (NMR) measurements on single crystals of three-dimensional (3D) perovskite FAPbI_3_.

FAPbI_3_ single crystals were fabricated following a previously published protocol.^40^ Briefly, a 1 M solution of formamidinium iodide (687.9 mg, 4 mmol; >99.99%, Greatcell Solar Materials) and lead­(II) iodide (1844.0 mg, 4 mmol; 99.99% trace metal basis, Tokyo Chemical Industries) in 4 mL of γ-butyrolactone (Alfa Aesar) was prepared. The solution was stirred at 60 °C for 4 h, then filtered with a 25 mm diameter, 0.45 μm pore glass microfibre filter. The filtrate was placed in a vial and heated in an oil bath undisturbed at 95 °C for 4 h until small crystals formed. The crystals were then dried in a vacuum oven at 180 °C for 45 min. All synthetic work besides drying was conducted in a N_2_ glovebox.

MAS NMR spectroscopy was carried out using a commercial Bruker Avance Neo 400 MHz spectrometer equipped with an LTMAS 3.2 mm Bruker ^1^H/X/Y triple-resonance probe. All measurements were conducted at approximately 95 K by using an 8 kHz MAS spin rate. For both ^13^C and ^15^N measurements, a ^1^H-X cross-polarization (CP) MAS pulse sequence was used. γ-Glycine was used to calibrate the ^1^H, ^13^C, and ^15^N radiofrequency field amplitudes (60, 40, and 140 kHz, respectively) and CP contact times (1 and 3 ms for ^1^H–^13^C and ^1^H–^15^N, respectively), and to reference ^13^C and ^15^N chemical shifts (174.9 ppm for ^13^C of C=O; 32.9 ppm for ^15^N). ^1^H decoupling at an RF field of 60 kHz was used during the acquisition in all measurements. We summarize the experimental parameters for all NMR measurements reported here in Table S3.

Immediately prior to measurement, the crystals were gently crushed and heated at 150 °C on a hot plate to ensure they were in the 3D FAPbI_3_ α-phase. These crushed crystals were packed inside a 3.2 mm sapphire rotor. This process was carried out in ambient air. The same packed rotor was used for all of the measurements reported here. The crystals were rapidly cooled (freeze) from 298 to 95 K at a rate of 5000–10,000 K min^–1^ by inserting the rotor into the probe at 95 K. Between each measurement, the crystals were rapidly warmed to 298 K by ejecting the rotor into ambient air, where it was kept for at least 5 min before the next cooling cycle. Notably, prior to the first measurements (freeze 1), the rotor had been cooled and heated in this manner several times. We therefore discount a difference between the first and subsequent quenching events as the source of observed ^15^N spectral differences between freeze 1, freeze 2, and freeze 3.

### Calculation of ^15^N Chemical Shifts

First-principles calculations of ^15^N chemical shifts were performed using DFT with the Quantum ESPRESSO^41,42^ package, employing the Perdew–Burke–Ernzerhof exchange–correlation functional and the gauge-including projected augmented wave method.^43,44^

Calculations were performed for two types of structures: the ground-state *a* ^–^ *b* ^–^ *b* ^–^ structure and three representative configurations of the cooled *a* ^–^ *a* ^–^ *c* ^+^ structure. In the latter case, atomic configurations for shielding calculations were extracted from molecular dynamics cooling simulations conducted in a 96-atom supercell. We set the plane-wave energy cutoff of 80 Ry for wave functions and 640 Ry for the charge density. We used a Γ-centered 2 × 2 × 2 *k*-point grid for Brillouin zone sampling.

To relate the computed trace of the shielding tensor σ_calc_ to experimental ^15^N chemical shifts δ_exp_, an empirical scaling was applied based on reference data.^45^ The scaling was performed via linear regression of computed shielding against experimentally measured chemical shifts from LGLUAC11, GLUTAM01, BITZAF, and CIMETD. This set corresponds to ten inequivalent local environments for N, spanning chemical shifts from −1.3 to 249.5 ppm.

The final chemical shifts were obtained using the linear transformation

1 $$\delta_{\mathrm{calc}}=a\cdot \sigma_{\mathrm{calc}}+b$$

where the parameters *a* and *b* of −1.05 and 201.88, respectively, were determined empirically from regression analysis of the reference data set.

### Dynamical Structure Factor from MD

We compute the dynamical structure factor from MD simulations using the dynasor package.^46^ For each structure prototype, we run 40 independent simulations, each 100 ps long, and average *S*(*q*, ω) over all the runs. The total *S*(*q*, ω) is given by the sum of the coherent and incoherent dynamical structure factors, which are weighted with their respective neutron scattering lengths. The resulting vibrational spectra are dominated by hydrogen motion due to its large incoherent scattering length. Since hydrogen dynamics is mostly *q* independent, we sum *S*(*q*, ω) over *q*-points between 0 and 15 rad/Å. The spectrum is calculated at 10 K, which means that the classical spectra obtained from MD do not capture the correct quantum statistics (intensities of the peaks). Therefore, we rescale the spectrum by

2 $$S_{\mathrm{QM}}\left(q,\omega \right)=\frac{\omega }{1-\exp\left(\hbar \omega /k_{B}T\right)}S\left(q,\omega \right)$$

as described in ref 47.

## Results

### Searching for the Lowest Energy Structure in FAPbI_3_

To understand the energy landscape of FAPbI_3_, we perform an extensive sampling of possible structures as shown in Figure 1. About a million initial structures are created in 2 × 2 × 2 supercells of the cubic primitive cell, incorporating randomized FA orientations and tilt modes with random mode amplitudes for each Cartesian direction. We relax each structure until the largest force on any atom falls below 0.1 meV Å^–1^. The resulting perovskite structures are then classified into Glazer structures^35^ by projection onto the M and R phonon modes (corresponding to octahedral tilting) as done in refs 32,48,49 . The ground-state (GS) perovskite structure is identified as *a* ^–^ *b* ^–^ *b* ^–^ with space group *C*2/*c* in the Glazer space as indicated in red in Figure 1A. Figure 1B shows the structure of *a* ^–^ *b* ^–^ *b* ^–^, where all FAs point in the same direction. The second lowest energy structure is identified as *a* ^0^ *b* ^–^ *b* ^–^ (space group *Imma*), which is structurally very similar to the ground state but lacks a small out-of-phase tilt around the *x*-axis. This similarity makes the two structures difficult to distinguish. We note that *a* ^–^ *b* ^–^ *b* ^–^ (or *a* ^0^ *b* ^–^ *b* ^–^) is not a common structure for halide perovskites; however, the preference for negative tilts can be a property related to the coupling between the FA molecule and the inorganic framework (see Table S2). A well-known example that adopts this ground state is the antiferroelectric PbZrO_3_,^50^ where the octahedral tilts are accompanied by an antipolar displacement pattern of the B-site cations. Interestingly, in our *a* ^0^ *b^–^b^–^* structure, we similarly observe displacements of the B-sites (Pb atoms), consistent with this antipolar motif, suggesting that the FAPI ground state likely could exhibit an antiferroelectric behavior. We also identify other possible structures with small energy differences, competing with the GS structure seen in Figure 1A. The atomic structures with preferred FA orientations of other relevant low-energy structures, i.e., *a* ^–^ *a* ^–^ *c* ^+^ (space group *Pnma*) and *a* ^0^ *a* ^0^ *c* ^+^ (space group *P*4/*mbm*), are shown in Figure 1C,D, respectively. We note that the space group assignments are purely done for the ideal perovskite using the Glazer notation. The total energies calculated using NEP and DFT are provided in Table S1, demonstrating good agreement with DFT calculations.

**Figure 1:**
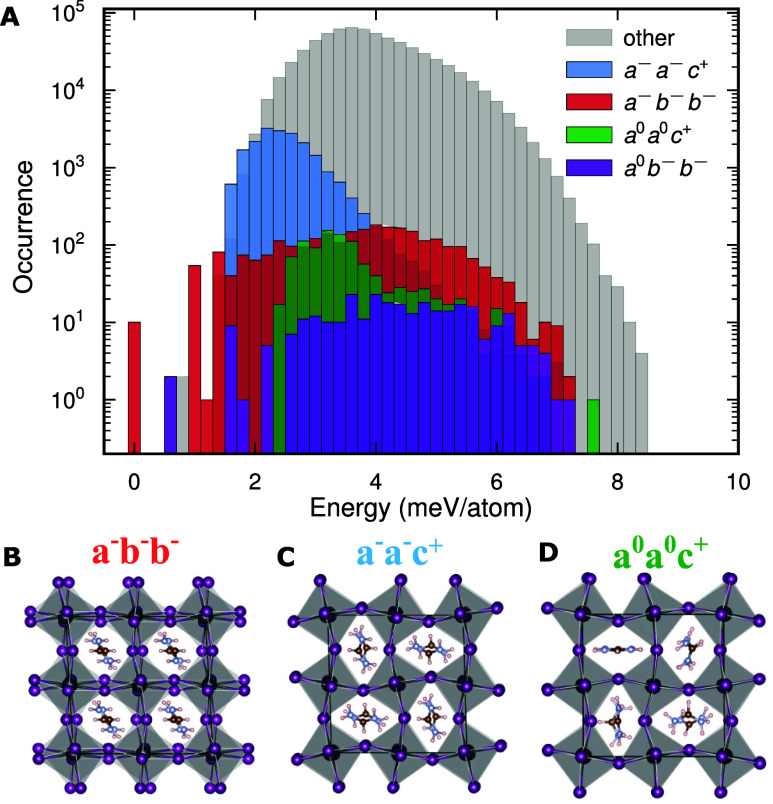
(A) Energy distribution of fully relaxed perovskite phases of FAPbI_3_ structures obtained by considering about a million different tilted structures with randomized FA orientations in 2 × 2 × 2 supercells of a corresponding primitive cell. Relevant low-energy structures are marked with color. Structural view of (B) *a* ^–^ *b* ^–^ *b* ^–^, (C) *a* ^–^ *a* ^–^ *c* ^+^, and (D) *a* ^0^ *a* ^0^ *c* ^+^ phases is shown.

### Behavior during Cooling and Heating

After identifying the most stable structure at 0 K, we now analyze heating and cooling runs to assess the phase transitions and compare them with experimental findings. Phase transitions can readily be seen as discrete or continuous changes in the thermodynamic properties like energy, heat capacity, and lattice parameters. To check the rate effects, we run simulations with different heating and cooling rates, using a supercell which is equivalent to a 16 × 16 × 16 repetition of primitive cubic (12-atom) cell and a 8 × 8 × 8 repetition of an *a* ^–^ *b* ^–^ *b* ^–^ (96-atom) cell. The convergence of the lattice parameter, energy, and heat capacity with respect to the heating and cooling rates can be found in Figure S3. Note that our simulations are conducted using supercells based on repetitions of the primitive cubic cell. The lattice parameters we plot are computed as the norms of the cell vectors in these supercells. Figure 2 shows the mentioned parameters as a function of temperature with the slowest heating and cooling rates (6.34 K ns^–1^). On heating, starting from the *a* ^–^ *b* ^–^ *b* ^–^ structure, the simulation yields a transition to β-phase at about 190 K and then to α-phase at about 315 K. In the cooling run, the simulation captures the same α to β transition; however, its transition into a different low-temperature phase occurs at about 120 K, which is 2 meV/atom higher in energy than the ground state. The low-temperature transition thus exhibits hysteresis and, in the heating run, appears to be of first-order in character. In contrast, the β to α-phase transition is a continuous one.

**Figure 2:**
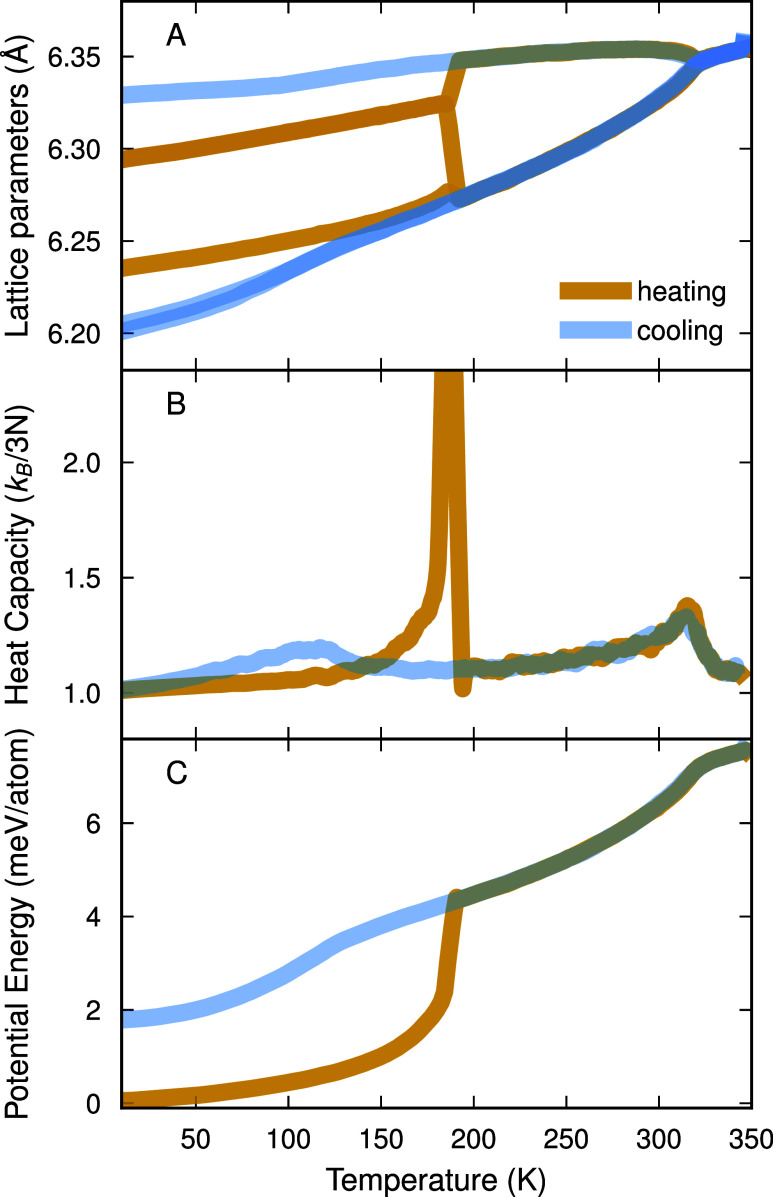
(A) Lattice parameters, (B) heat capacity, and (C) energy from heating and cooling MD with 6.34 K/ns rate, respectively, in FAPbI_3_.

Here, it is interesting to note again that the low-temperature structure obtained from cooling in experiments is not fully understood.^5,7,12,15,20,22,24^ To determine whether the structure found in our cooling simulations corresponds to the one encountered in experimental studies, we therefore analyze it in more detail.

### Tilt Angle Analysis

To gain additional insight into the low-temperature phase obtained from the cooling run, we first focus on the octahedral tilting patterns of the system at different temperatures. Here, we compute the PbI_6_ (see Figure S7a) octahedral tilt angles in the perovskite structures during cooling MD simulations as done in refs 17 and 51. First, the PbI_6_ octahedron is matched to a fully symmetric octahedron in an ideal cubic perovskite following the algorithm in ref 52 as implemented in ovito,^53^ which generates the rotation and scales for optimal mapping. Functionality from the scipy package^54^ is used to convert the rotation to Euler angles (see Figure S7b for the definition of the Euler angles). Following Glazer’s approach,^35^ we chose the rotation that produces the angles in increasing magnitude among the three possible options.

The distribution of octahedral tilt angles over the entire temperature range from the cooling run is shown in Figure 3. The transition temperatures obtained from Figure 2 are indicated by vertical dashed lines. In the high-temperature α-phase, which can be described as *a* ^0^ *a* ^0^ *a* ^0^ in Glazer notation, the tilt angle distributions are monomodal and centered at around 0°. Next, in the β-phase, the ψ angle, which characterizes the tilt in the *z* direction, obtains an average value of about 10°, which, upon visual inspection with ovito, can be identified as an in-phase tilting pattern. Glazer notation thus describes this β phase as *a* ^0^ *a* ^0^ *c* ^+^. The tilt angles θ and ϕ become nonzero in the low-temperature γ phase. After analysis of tilt patterns in all directions, we found that the *c* ^+^ tilt from the *a* ^0^ *a* ^0^ *c* ^+^ structure becomes more robust with an average value of about 15 ° in the γ-phase. Additional out-of-phase tilt with a value of θ = ϕ ≃5° appears along the *x* and *y* directions. Thus, one can characterize this γ-phase as *a* ^–^ *a* ^–^ *c* ^+^ in Glazer space. The snapshots obtained from the cooling simulation run, highlighting representative temperatures and corresponding octahedral tilt configurations, are shown in Figure S8. It is important to note that another similar FA-based perovskite FAPbBr_3_ structure below 153 K has also been experimentally identified as the same *a* ^–^ *a* ^–^ *c* ^+^ (*Pnma*) phase.^55–59^

**Figure 3:**
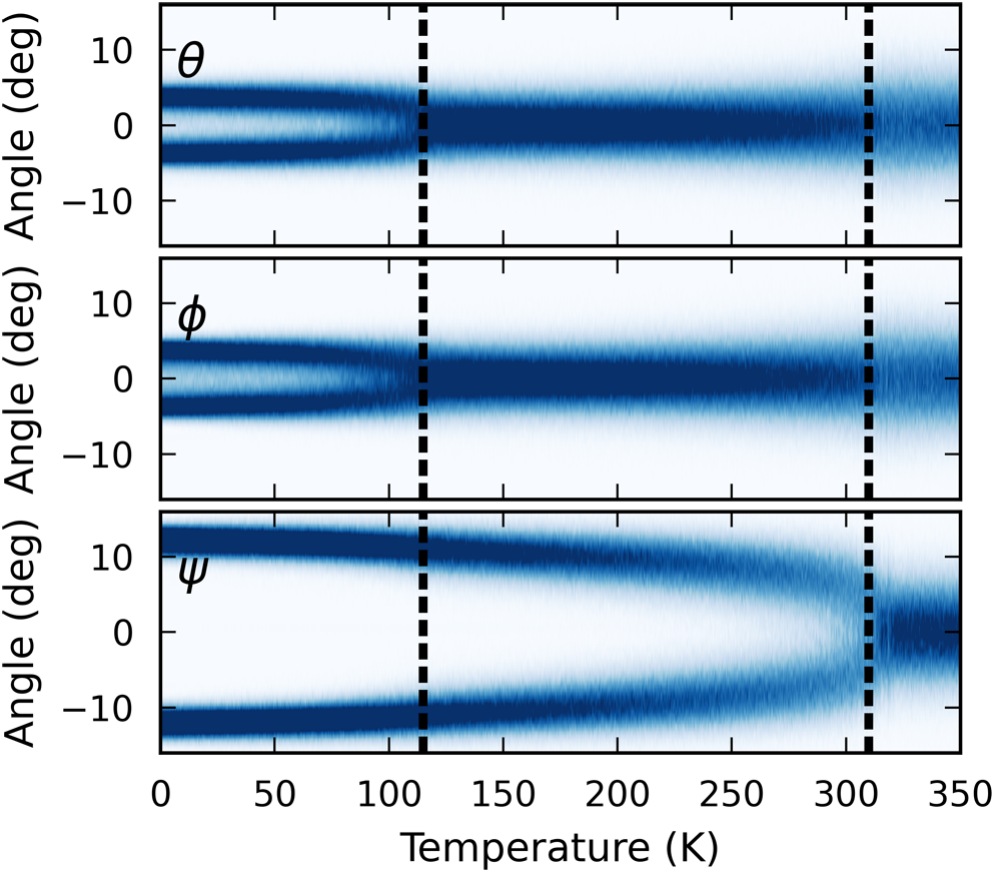
Maps of tilt angles as a function of the temperature from cooling MD runs. Dashed black lines represent two successive phase transitions from *a* ^0^ *a* ^0^ *a* ^0^ to *a* ^0^ *a* ^0^ *c* ^+^ and *a* ^0^ *a* ^0^ *c* ^+^ to the *a* ^–^ *a* ^–^ *c* ^+^-phase.

As noted earlier, the structure we find upon cooling does not correspond to the GS structure of FAPbI_3_ identified in section (Figure 2). This suggests two possibilities: (i) our MD simulations do not reach the true low-temperature structure of FAPbI_3_ (*a* ^–^ *b* ^–^ *b* ^–^) due to limitations in cooling rates, whereas experiments do, or (ii) the *a* ^–^ *a* ^–^ *c* ^+^ structure represents a frozen metastable state, mirroring a physical scenario where FAPbI_3_ remains kinetically trapped during cooling instead of transitioning to the GS structure, which is also the case in experiments. To test these hypotheses, we analyze the ordering and dynamics of FA molecules and compare simulated characteristics of the potential phases to experimental measurements.

### Ordering of FAs

To understand the local symmetry, we look at the molecule reorientation in different phases of FAPbI_3_. We consider the vector connecting the two N atoms, **r**_NN_, and the vector between C and H atoms, **r**_CH_, in a FA molecule as shown in Figure S7c. We compute the orientation represented by polar angle ϕ and azimuthal angle θ for each of them. ϕ is the angle between *r*_NN_ (*r*_CH_) and *z* direction, and θ denotes the angle in the xy-plane. Figure 4 represents the probability distributions over θ and ϕ (*P* (θ,ϕ)) for N–N and C–H vectors in the three different phases of FAPbI_3_ from the cooling run.

**Figure 4:**
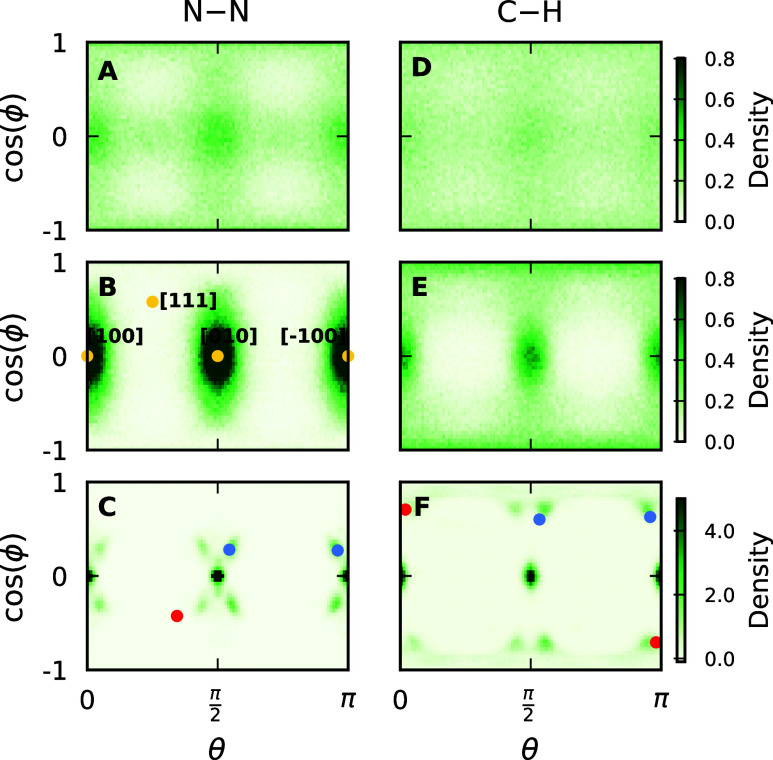
Probability distribution *P* (θ, ϕ) of N–N vectors (A–C), and of C–H vectors (D–F) in *a* ^0^ *a* ^0^ *a* ^0^, *a* ^0^ *a* ^0^ *c* ^+^, and *a* ^–^ *a* ^–^ *c* ^+^ phases (top to bottom), respectively. This *a* ^–^ *a* ^–^ *c* ^+^ phase is obtained from the cooling run. Here, θ refers to the angle in the *x*–*y* plane, and ϕ is the angle to the *z*-axis. The position of the vectors in Cartesian coordinates is marked in yellow in (B). The orientation of the N–N and C–H vectors of FAs in an ideal *a* ^–^ *a* ^–^ *c* ^+^ phase and *a* ^–^ *b* ^–^ *b* ^–^ phase is shown by the blue and red dots in (C, F), respectively.

In the high-temperature *a* ^0^ *a* ^0^ *a* ^0^ phase (at 330 K), the N–N and C–H vectors are homogeneously distributed, indicating an almost-free molecular rotation of FA molecules as shown in Figure 4A,D. Once cooled down from the *a* ^0^ *a* ^0^ *a* ^0^ to the *a* ^0^ *a* ^0^ *c* ^+^-phase (at 200 K), we notice a pattern appearing in the distributions, which is symmetric in the *xy*-plane as shown in Figure 4B,E, also observed by Tua et al.^7^ The N–N vectors are most likely to be aligned with the *x* ([100]) and *y* ([010]) directions. This arrangement of FA molecules in Figure 4B,E is also reflected in the 2 × 2 × 2 supercell of the *a* ^0^ *a* ^0^ *c* ^+^ structure. The pattern is not as clear for C–H compared to N–N; however, it shows some preferred orientations along [100], [010], and [001] directions.

The distribution becomes sharper and changes again when cooled into the *a* ^–^ *a* ^–^ *c* ^+^ phase (at 10 K) for both N–N and C–H vectors (Figure 4C,F). The distributions mostly retain the preferred orientations from the *a* ^0^ *a* ^0^ *c* ^+^ phase, but with four symmetric additional orientations appearing as ”wings”. These wings correspond to the orientations found in the ideal *a* ^–^ *a* ^–^ *c* ^+^ structure (shown in Figure 1C), as marked by the blue dots in Figure 4C,F. However, the cooled structure differs significantly from the ideal one, as it still has a large proportion of FA molecules stuck in the orientations characteristic of the *a* ^0^ *a* ^0^ *c* ^+^ phase. Note here that the four symmetric “wings” each correspond to a symmetrically equivalent version of the *a* ^–^ *a* ^–^ *c* ^+^ structure.

We also compare the FA orientations to those of the GS phase, represented as red dots in Figure 4C,F. This orientation corresponds to a very low probability distribution at temperatures close to the transition. Therefore, a significant free energy barrier likely prevents the FA molecules from aligning as in the GS phase, leading to structural freezing in a metastable state. To have a quantitative picture, we estimate that the free energy barrier for N–N vectors to align like in the GS phase using *F* = −*k*_B_  ln­(*P*(θ,ϕ)), where *k*_B_ is the Boltzmann constant, and find it to be more than 100 meV per FA at 200 K (see Figure S9).

Next, we assess the ordering of FA molecules in different relevant structures. This is done by analyzing the nearest neighbor correlation of N–N and C–H vectors as shown in Figure S10 at different temperatures. The results highlight that the *a* ^–^ *a* ^–^ *c* ^+^ structure found upon cooling is significantly more disordered than the ideal *a* ^–^ *a* ^–^ *c* ^+^ and *a* ^–^ *b* ^–^ *b* ^–^ phases. Notably, the ideal *a* ^–^ *a* ^–^ *c* ^+^ phase loses its strong ordering (and approaches that of the cooled structure) when heated up to only 50 K, whereas the ground state, *a* ^–^ *b* ^–^ *b* ^–^, remains very ordered, indicating FAs are more locked into place in this phase.

These analyses of the FA orientational distributions and ordering demonstrate that the cooled structure has several different local FA orientations and environments, indicating more disorder compared to that of the ideal structures. This is qualitatively in agreement with experimental studies, which observe a significant degree of disorder in the low-temperature structure.^12,20^

### Rotational Dynamics of FAs

Next, we analyze the rotational dynamics of FA molecules by calculating the orientational autocorrelation function (ACF) as defined in

3 $$C\left(\tau \right)=\frac{\left\langle r_{\mathrm{NN}}^{i}\left(t\right)r_{\mathrm{NN}}^{i}\left(t+\tau \right)\right\rangle }{\left\langle r_{\mathrm{NN}}^{i}\left(t\right)r_{\mathrm{NN}}^{i}\left(t\right)\right\rangle }$$

where **r**_NN_^*i*^(*t*) (**r**_CH_^*i*^(*t*)) is the N–N (C–H) bond vector at time *t* for the *i*th FA molecule. To this end, we run MD simulations at several temperatures starting from the phase corresponding to those temperatures. The N–N (C–H) bond vector **r**_NN_ (**r**_CH_) of each FA unit is sampled in the NVE ensemble for 1000 ps (at the volume previously obtained from NPT runs). Figure 5A,B represent the ACF of the N–N and C–H axes as a function of time, respectively. The ACF decays faster at high temperature, reflecting faster reorientation of the FA molecules in the high-temperature phase. However, it decays more slowly with decreasing the temperature, indicating freezing of FA molecules.

**Figure 5:**
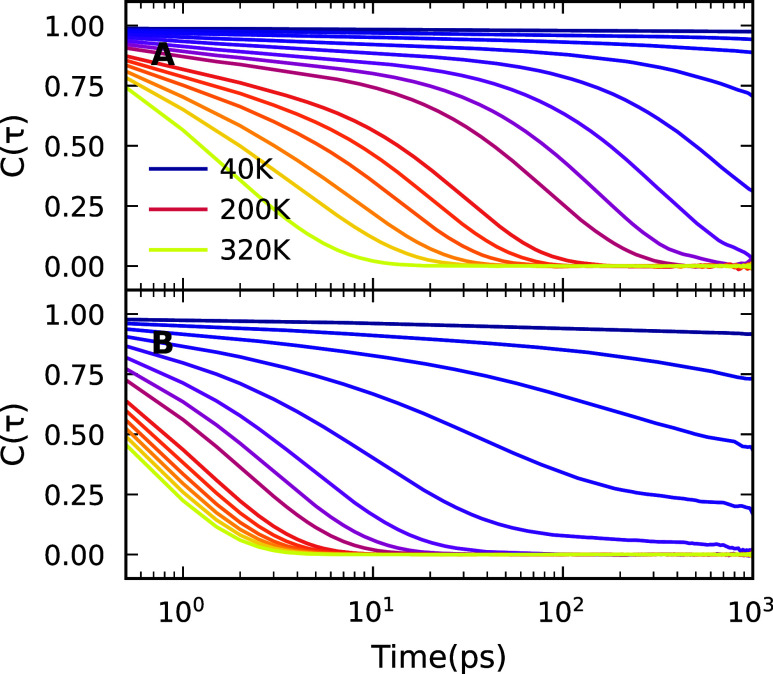
Autocorrelation function *C* (τ) for the orientation of (A) N–N and (B) C–H vectors in FA units. The spacing between the lines is 20 K.

The decay in the ACF can be modeled with an exponential function as *C*(τ) = *A*_1_ *e* ^–τ/τ_rot_^ + *A*_2_ *e* ^–τ/τ_vib_^, where *A*_1_ and *A*_2_ are prefactors, τ_rot_ denotes the rotational times, and τ_vib_ accounts for vibrations of the FA molecule.^16,60^ The rotational times of the N–N and C–H vectors are shorter for the tetragonal *a* ^0^ *a* ^0^ *c* ^+^ phase (above 120 K) than those of the low-temperature phase (below 120 K). Figure 6 displays the estimation of the rotational time of N–N and C–H vectors. The rotational times of the C–H axis measured in experiment^12^ are in reasonable agreement with our predicted values. The offset between the present study and experiment can possibly be attributed to the model accuracy and difficulties in capturing the slow dynamics of FA molecules in MD.

**Figure 6:**
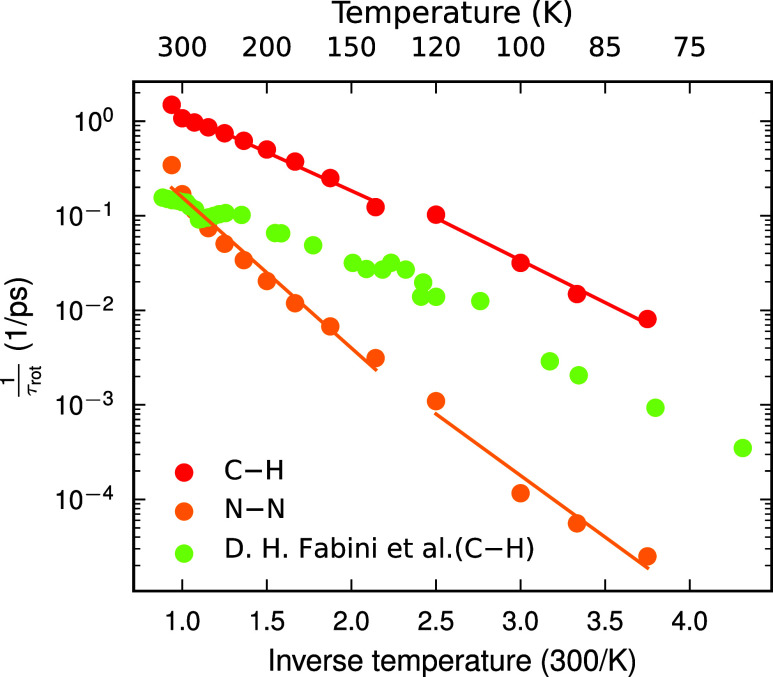
Rotation rate (1/τ_rot_) as a function of the temperature. The solid lines correspond to Arrhenius fits mentioned in the text. Green symbols represent the data for the rotation of C–H vectors from the experiment for comparison.^12^

Subsequently, we model the temperature dependence of the rotational time using the Arrhenius eq 1/τ_rot_∝*e* ^–*E*_A_/*k*_B_ *T*^, where *E*_A_ is the activation energy and *k*_B_ is the Boltzmann constant, which fits the data well. This yields the activation barrier of the rotational process for different phases, which are provided in Table 1 along with a comparison with the literature. We find good agreement with experimentally measured and calculated values from ref 12. Furthermore, we note that the barrier for the N–N vector in the *a* ^0^ *a* ^0^ *c* ^+^ phase, 94.9 meV, is consistent with the barrier obtained from the free energy landscapes at 200 K (Figure S9).

**Table 1:** Activation Energy Barriers in meV for Molecular Rotation of the N–N and C–H Axes from the Present Study and the Available Literature^12^ for the *a* ^0^ *a* ^0^ *c* ^+^ (*β*-Phase) and *a* ^–^ *a* ^–^ *c* ^+^ (*γ*-Phase) Phases of FAPbI_3_

	**a** ^0^ **a** ^0^ **c** ^+^ (β-phase)		**a** ^–^ **a** ^–^ **c** ^+^ (γ-phase)	
	C–H	N–N	C–H	N–N
experiment^12^	45	–	84	–
DFT^12^	39	–	63	–
NEP	48.5	94.9	53.3	77.5

Lastly, we compare the dynamics of FAs obtained above with the GS structure (see Figure S12). Interestingly, the ACF in the GS phase indicates that all of the FA molecules are frozen with *C*_*i*_(τ)∼1 throughout the time range (10 ns) and up to 120 K. A rough estimate of the rotational time for the very flat ACF at 120 K in the GS is at least 20 μs. This suggests that the FAs in this phase do not rotate, unlike in the experimentally observed low-temperature phase, where they rotate on a nanosecond time scale at these temperatures. This indicates that the experimental phase does not reach the ground-state structure and that the kinetic trapping observed in our simulations reflects a physically realistic metastable state.

### Experimental Verification

To further validate the low-temperature phase found in simulations, we compare our results with magic angle spinning nuclear magnetic resonance (MAS NMR) spectroscopy and inelastic neutron scattering (INS) experiments at 95 and 10 K, respectively. The NMR spectra provide insight into the local environment of FA in the γ-phase, revealing structural changes upon repeated freeze–thaw cycles. While the ^13^C spectra are identical for each of the three freeze–thaw cycles (Figure 7A), the ^15^N spectra show a distribution of several overlapping signals with slight differences in their relative population between each cycle (Figure 7B), suggesting that the local structure can change in each freezing event. To better understand the origin and variability of the ^15^N line shape, we perform chemical shield calculations (Figure 7C). These calculations are carried out on the ground-state *a* ^–^ *b* ^–^ *b* ^–^ structure and the cooled *a* ^–^ *a* ^–^ *c* ^+^ structure. In the ordered *a* ^–^ *b* ^–^ *b* ^–^ phase, all N atoms are equivalent, resulting in a single chemical shift value. In contrast, the disordered cooled structure exhibits a broad distribution of ^15^N chemical shifts due to variations in the local environment. We note that our calculations systematically underestimate the absolute chemical shift values compared to the experiment, which is expected, as they do not include spin–orbit coupling effects. Additionally, since they are performed in rather small supercells, they do not exactly reflect the distribution of FA orientations. Nevertheless, our calculations qualitatively demonstrate that the experimentally observed distribution of ^15^N chemical shifts can only be explained by cation disorder, as found in the cooled structure. This also allows us to again rule out the ordered ground-state structure as that present in the experiments.

**Figure 7:**
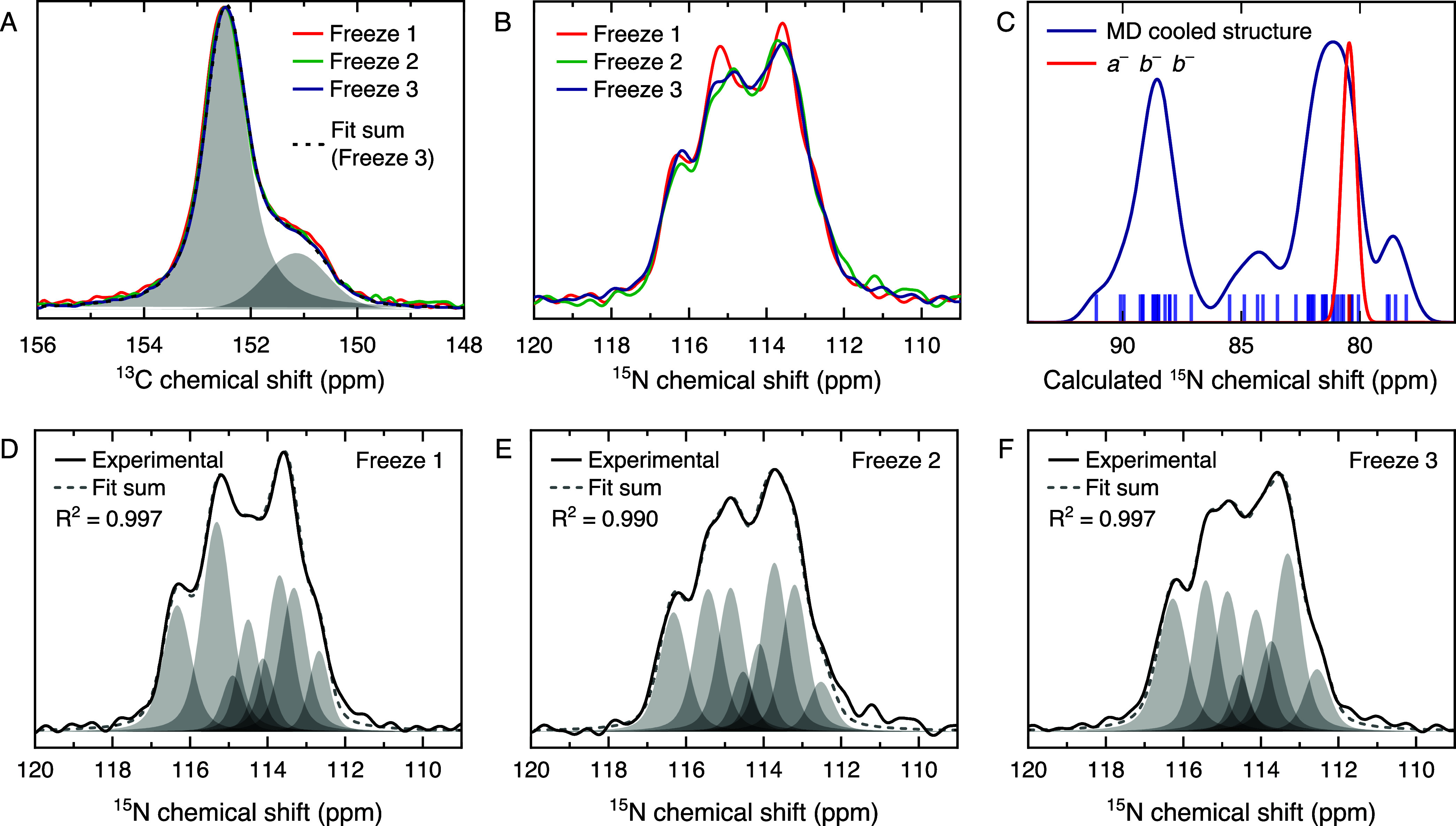
Low-temperature ^1^H–^13^C (A) and ^1^H–^15^N (B) cross-polarization MAS NMR spectra (9.4 T, 8 kHz) of 3D FAPbI_3_ single crystals acquired at 95 K during three sequential freeze–thaw cycles. (C) Calculated ^15^N chemical shift distribution for 3D FAPbI_3_ at 95 K. (D–F) Fitting of ^15^N spectra from each freeze event demonstrating that overall spectra are the cumulative result of varying the population of eight distinct signals.

Precise fitting of the experimental ^15^N NMR spectra is challenging due to the presence of distinct but poorly resolved signals. Nevertheless, we are able to fit eight Gaussian peaks to all three ^15^N spectra, whose relative populations vary from cycle to cycle (Figure 7D–F) but for which the same ^15^N chemical shift (±0.05 ppm) for each peak is maintained across all three cycles. These fitted signals should be considered a guide to the eye only; nonetheless, this small number of well-defined and experimentally reproducible FA local environments at 95 K is consistent with the result of our MD run, where we found that in the low-temperature phase, the N–N and C–H vectors point in a limited number of directions.

We next compare the *a* ^–^ *a* ^–^ *c* ^+^ structure, identified in our simulations as the best representation of the low-temperature γ phase of FAPbI_3_, to the experimental data previously reported in the literature. Single-crystal X-ray diffraction on the γ phase has been challenging because around 100 K the Bragg peaks substantially broaden and split, leading to many unindexed reflections and preventing structure refinement.^61^ On the other hand, structural information on this low-temperature phase can also be accessed through the vibrational signatures of FA obtained in inelastic neutron scattering (INS) experiments.^14^ Here, we compare the vibrational spectra computed for our *a* ^–^ *b* ^–^ *b* ^–^ structure, the ideal *a* ^–^ *a* ^–^ *c* ^+^ structure, and the *a* ^–^ *a* ^–^ *c* ^+^ structure obtained from MD runs to the experimental data (Figure 8). The INS data presented in this study were collected using the TOSCA instrument for a pure FAPbI_3_ sample from ref 15. Note here that the simulated spectra are without any extra broadening that would be present in the experiment, for TOSCA, this broadening is about 0.06–0.3 meV.^62^

**Figure 8:**
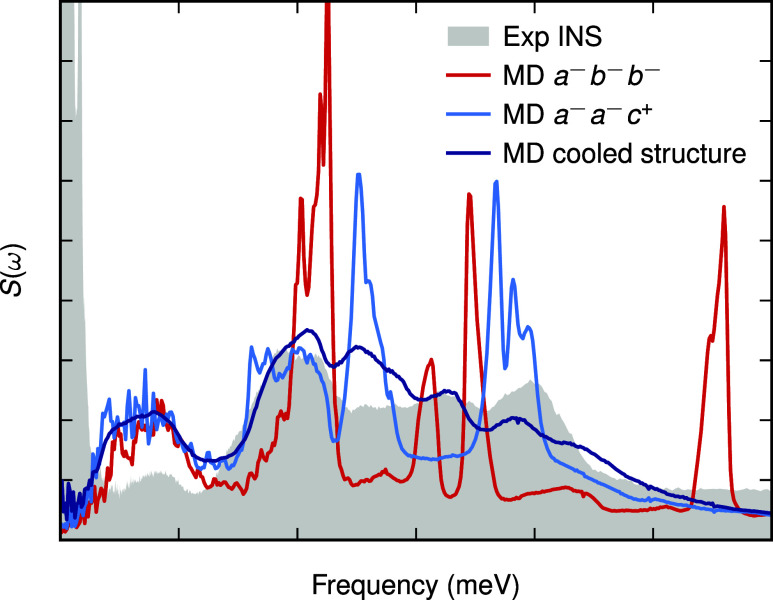
Simulated inelastic neutron scattering spectra, *S*(*q*, ω), for three different structures compared to the experimental spectra from ref 14 at 10 K. Here, *a* ^–^ *b* ^–^ *b* ^–^ and *a* ^–^ *a* ^–^ *c* ^+^ refer to the structures found from the ground-state search carried out in Figure 1, and the cooled structure refers to the structure found upon cooling. The experimental and simulated spectra are scaled with an arbitrary constant to make them appear on the same scale.

We compute the dynamical structure factor, which is dominated by hydrogen motion due to its large incoherent scattering length. We find that the spectra obtained from MD simulations starting from the ideal *a* ^–^ *b* ^–^ *b* ^–^ and *a* ^–^ *a* ^–^ *c* ^+^ structures contain sharp peaks, whereas the spectrum for the structure found upon cooling is substantially broader and agrees well with the experimental spectrum. This is likely due to the uniform FA ordering and local environments in the ideal structures, which result in sharp peaks, whereas the more disordered cooled structure exhibits a broader spectrum due to the presence of multiple distinct hydrogen environments. Notably, the broader features observed in our simulated INS spectra also compare well with those reported by Drużbicki et al., where the INS spectrum of pure FAPbI_3_ exhibits similar broad features, consistent with a disordered phase.^14^ This agreement suggests that the *a* ^–^ *a* ^–^ *c* ^+^ structure obtained from the cooling run closely resembles the experimentally observed low-temperature γ phase. Therefore, we conclude that our model likely provides an accurate atomic-level description of the disordered γ phase.

## Discussion

The insights gained from our analysis shed light on the low-temperature phase of FAPbI_3_. MD simulations are inherently limited by time scale and computational constraints, often resulting in faster cooling rates and inadequate sampling of the energy landscape. This limitation frequently leads to kinetic trapping at local minima. For instance, while the true GS of MAPbI_3_ is the orthorhombic *a* ^–^ *a* ^–^ *c* ^+^ phase, conventional cooling MD simulations result in the intermediate tetragonal *a* ^0^ *a* ^0^ *c* ^–^ phase persisting down to 0 K. However, in the case of MAPbI_3_, it is established that the ground state is reached in experiments. For FAPbI_3_, existing literature remains inconclusive about the low-temperature phase, highlighting the need for further clarification. We identify the *a* ^–^ *a* ^–^ *c* ^+^ phase as a local minimum below 120 K in cooling MD runs and argue that freezing into that structure likely also occurs in experimental studies. Our study identifies the *a* ^–^ *b* ^–^ *b* ^–^ (or *a* ^0^ *b* ^–^ *b* ^–^) phase as the GS and investigates the factors that might prevent the system from transitioning to this state. Specifically, we analyze two components that can influence the system’s behavior: (i) the inorganic framework, focusing on tilting patterns and (ii) the organic framework, analyzing the orientation preferences and rotational dynamics of FA molecules.

The transition from the tetragonal *a* ^0^ *a* ^0^ *c* ^+^ phase to the orthorhombic *a* ^–^ *b* ^–^ *b* ^–^ GS requires switching from in-phase to out-of-phase tilts relative to the *c*-axis. This transition involves an energy barrier that likely stabilizes the *a* ^–^ *a* ^–^ *c* ^+^ phase by preserving the in-phase tilt along the *c*-direction. Focusing on the organic part of the system, FA molecules exhibit distinct behaviors in different phases. In the GS *a* ^–^ *b* ^–^ *b* ^–^ phase, the FA molecules are highly ordered, as evidenced by sharp peaks in the simulated inelastic neutron spectra (Figure 8). The cooled *a* ^–^ *a* ^–^ *c* ^+^ phase exhibits significant disorder, also reflected in the broader peaks in its NMR and INS spectra (Figures 7 and 8), which closely resemble experimental results and align with the observed structure in experiments. The observed disordered γ-phase, characterized by broader features in the simulated INS and NMR spectra, aligns well with the previous INS,^14^ neutron diffraction,^20^ and NMR studies,^12^ all of which indicate the formation of an orientational glass. In this context, we note that, at low temperatures, FAPbBr_3_ also exhibits a disordered phase, as reported in several studies.^25,56–59^ This observation has been further supported by Reuveni et al.,^63^ who used terahertz-Raman spectroscopy to demonstrate intrinsic local disorder existing in the *Pnma* orthorhombic structure by showing the broad peaks. Mozur et al. attributed this low-temperature disorder to frustrated interactions between elastic dipoles created by the electrostatic interactions of strongly quadrupolar FA cations with their surrounding anions. This quadrupolar interaction leads to T-shaped arrangements of FA molecules within planes.^57,58^ We observe a similar arrangement in the *a* ^0^ *a* ^0^ *c* ^+^ and *a* ^–^ *a* ^–^ *c* ^+^ phases (see Figure S8), suggesting that the underlying mechanism for frozen disorder in these systems is similar.

Moreover, transitioning from the *a* ^–^ *a* ^–^ *c* ^+^ phase to the GS requires the FA molecules to overcome an additional energy barrier exceeding 100 meV atom^–1^ to adopt the ordered orientation of the GS phase. This lower bound for the energy barrier associated with reorienting a single molecule toward the ground-state configuration helps explain why the structure remains trapped in a metastable phase. However, the full transformation would require collective molecular and octahedral reorientations as well as nucleation and growth of the ground-state phase, which present additional significant kinetic barriers. This observation is corroborated by the extended rotational relaxation times of FA molecules at lower temperatures (Figure 6), indicating a ”freezing” effect. Thus, the freezing of FA molecules appears to be an intrinsic feature of FAPbI_3_, locking the system in the metastable *a* ^–^ *a* ^–^ *c* ^+^ phase.

This freezing behavior is consistent with experimental evidence for glass-like dynamics in FAPbI_3_. Fabini et al. estimate the glass transition temperature to be near 50 K based on dielectric loss spectroscopy.^19^ However, the slow dynamics at such low temperatures are difficult to probe with MD simulations due to the time-scale limitations inherent to the method. Nonetheless, the qualitative behavior we observe supports the picture of a disordered frozen low-temperature state exhibiting orientational glass-like dynamics. We note that such glassy behavior has also been reported in mixed-cation or mixed-halide perovskites, where compositional disorder is commonly invoked as the origin of frustration.^64–67^ The fact that a similar behavior emerges in pure FAPbI_3_ underscores the intrinsic nature of the orientational disorder. This disordered low-temperature effect might explain some of the uncertainties in experimental studies of FAPbI_3_ and highlights the local structural variability and the complexity of its underlying dynamics.

## Conclusions

In conclusion, we used an interatomic machine-learned potential to investigate phase transitions and the dynamics of FA cations, aiming to clarify the low-temperature phase of FAPbI_3_.

The low-temperature phase of FAPbI_3_ has often been described as disordered, but this characterization lacks precision. Our study provides a detailed atomistic model, demonstrating that this disorder is not random. Instead, FA cations exhibit structured arrangements, such as in-plane T-shaped N–N vector correlations and frustrated C–H vector orientations. This partial order provides a more accurate depiction of the phase than simplistic “disorder” models. Furthermore, our tilt-angle analysis identifies the low-temperature phase as *a^–^a^–^c^+^* . We suggest that this particular structure is favoured due to the persistence of the *c* ^+^ tilt with small negative tilts developing along the other axes. Such a scenario that aligns with perovskite group–subgroup relationships and requires no or lower energy barrier to cross compared with other potential phases. Lastly, our model reproduces broad INS and NMR spectra in excellent agreement with experiments, confirming that the partial order we describe is consistent with observed structural dynamics. Together, these findings present a comprehensive and validated picture of the low-temperature phase. We believe that this work provides new insights into the low-temperature phase of FAPbI_3_, offering a detailed explanation of FA dynamics and the factors influencing kinetic trapping. Our findings help resolve existing ambiguities in the literature and advance our understanding of the structural and dynamic complexities of this material.

## Supplementary Material



## Data Availability

The raw NMR data and NEP relaxed structures are available on Zenodo: https://zenodo.org/records/16805881, DOI: 10.5281/zenodo.16805881

## Notes

The loss and RMSE during training, parity plots (NEP vs DFT), convergence of heating/cooling rate and finite-size effect, evolution of volume and angles between three cell vectors during heating/cooling, additional figures showing the illustration of octahedral tilt angles, snapshots of different phases appeared during cooling, free energy landscape of N–N vectors, nearest neighbor correlation analysis, ACF of each FA molecule in the system over time, details of determination of NMR cooling rate, total energies comparison of different phases between DFT and NEP, comparison between different exchange–correlation functionals, experimental parameters for solid-state NMR measurements (PDF)
