# Supplementary material for: Revealing the Low-Temperature Phase of FAPbI3 Using a Machine-Learned Potential
Source: J Am Chem Soc. 2025 Aug 14;147(41):37019–29. doi: 10.1021/jacs.5c05265 (PMC12532299; doi:10.1021/jacs.5c05265)
Supplement: Supplementary file 1 [file ja5c05265_si_001.pdf]

## Supporting Information

# Revealing the Low Temperature Phase of $FAPbI_3$ Using a Machine-Learned Potential

Sangita Dutta[1,*], Erik Fransson[1], Tobias Hainer[1], Benjamin M. Gallant[2], Dominik J. Kubicki[2], Paul Erhart[1], and Julia Wiktor[1,*]

[1]Department of Physics, Chalmers University of Technology, SE-41296, Gothenburg, Sweden
[2]School of Chemistry, University of Birmingham, Edgbaston, B15 2TT, United Kingdom

* sangita.dutta@chalmers.se; julia.wiktor@chalmers.se

## Supplementary Figures

- **Loss and RMSE during training of the NEP potential**

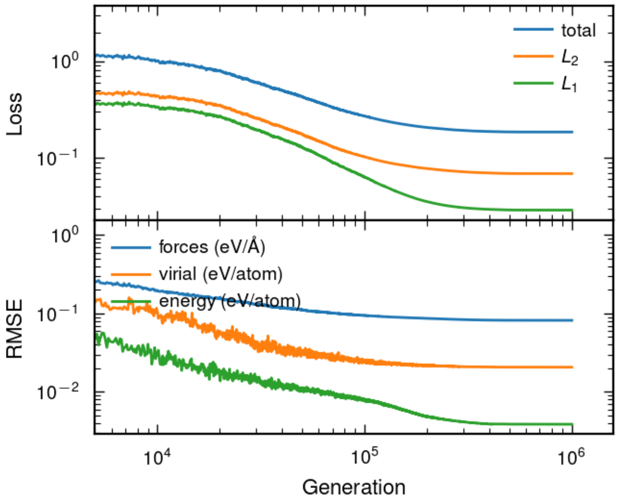


**Fig. S1:** Evolution of total loss as well as its individual contributions during training of the full model for $FAPbI_3$ based on the SCAN-VV10 functional. $L_1$, $L_2$: contribution from $L_1/L_2$-norm of parameter vector.

- **Parity plots of energy, force, and virial predicted by NEP versus DFT**

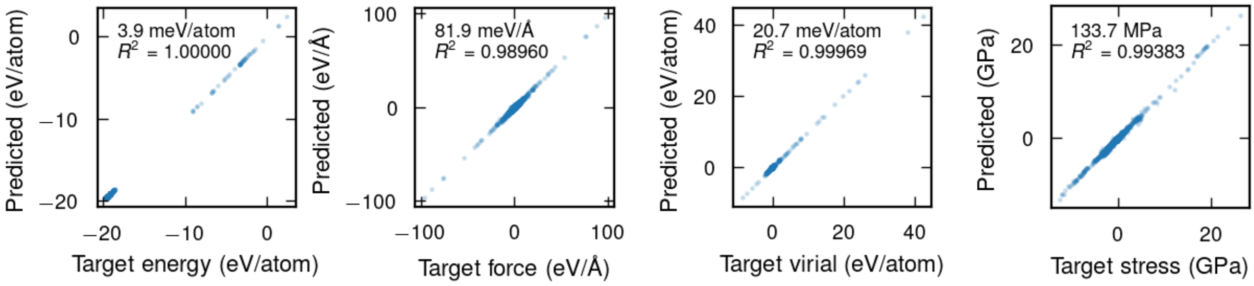


**Fig. S2:** Parity plots for total energies, forces, virials, and stresses for full model for $FAPbI_3$ based on the SCAN-VV10 functional.

- **Effect of different cooling and heating rates on potential energies, lattice parameters, and heat capacities**

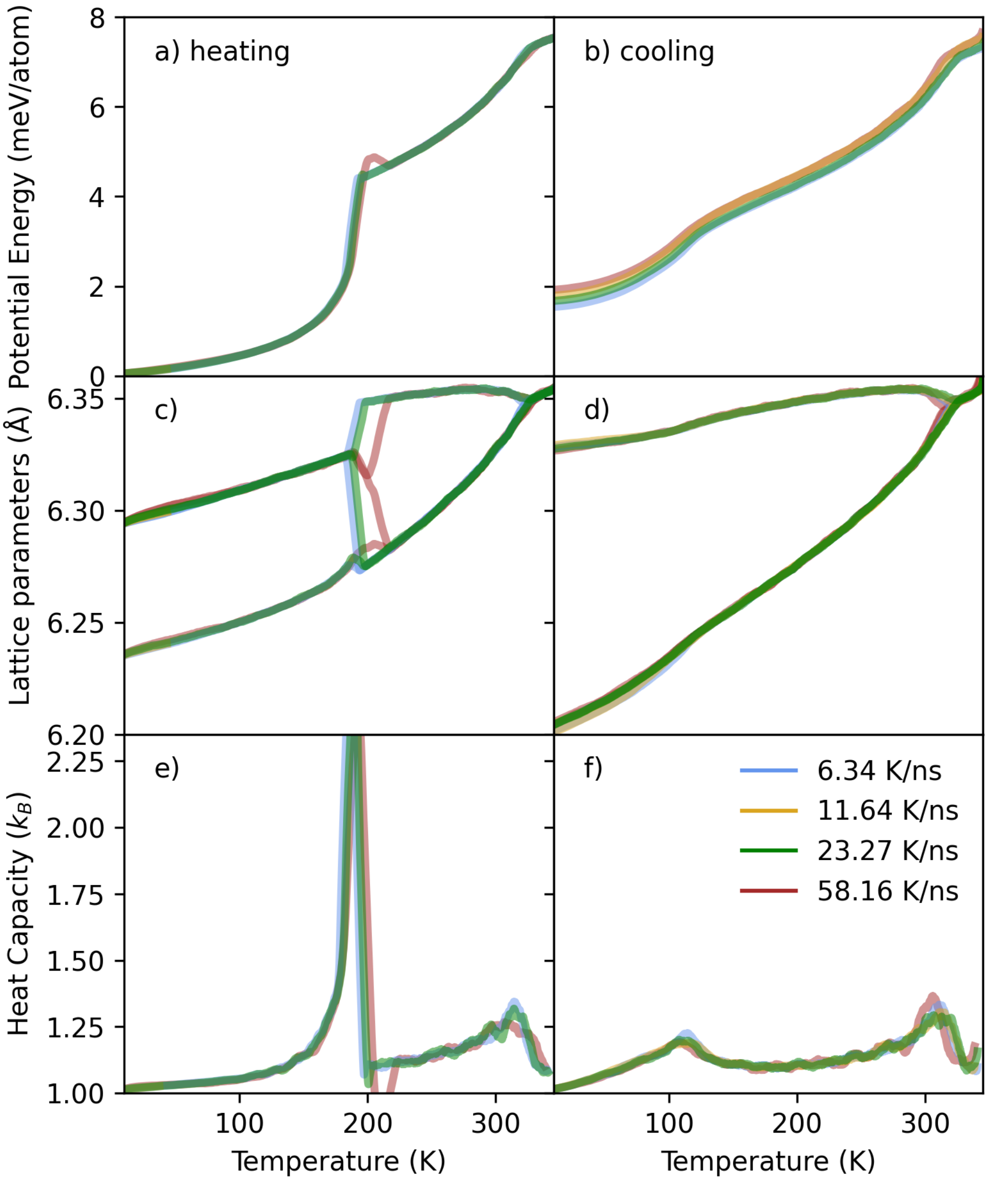


**Fig. S3:** (a) Potential energy, (b) lattice parameters, and (c) heat capacity, respectively in $FAPbI_3$ with different cooling rates. The energy difference between the structures with the highest and lowest cooling rates is 0.38 meV/atom.

- **Finite-size effect on potential energies, lattice parameters, and heat capacities**

To ensure the robustness of our results and address the impact of finite-size effects, we performed simulations on systems of varying sizes. Specifically, we considered systems with cell sizes of 12000 atoms, 20736 atoms, 32928 atoms, and 49152 atoms. The phase transition behaviours observed in these simulations were consistent across all system sizes, confirming the reliability of our findings.

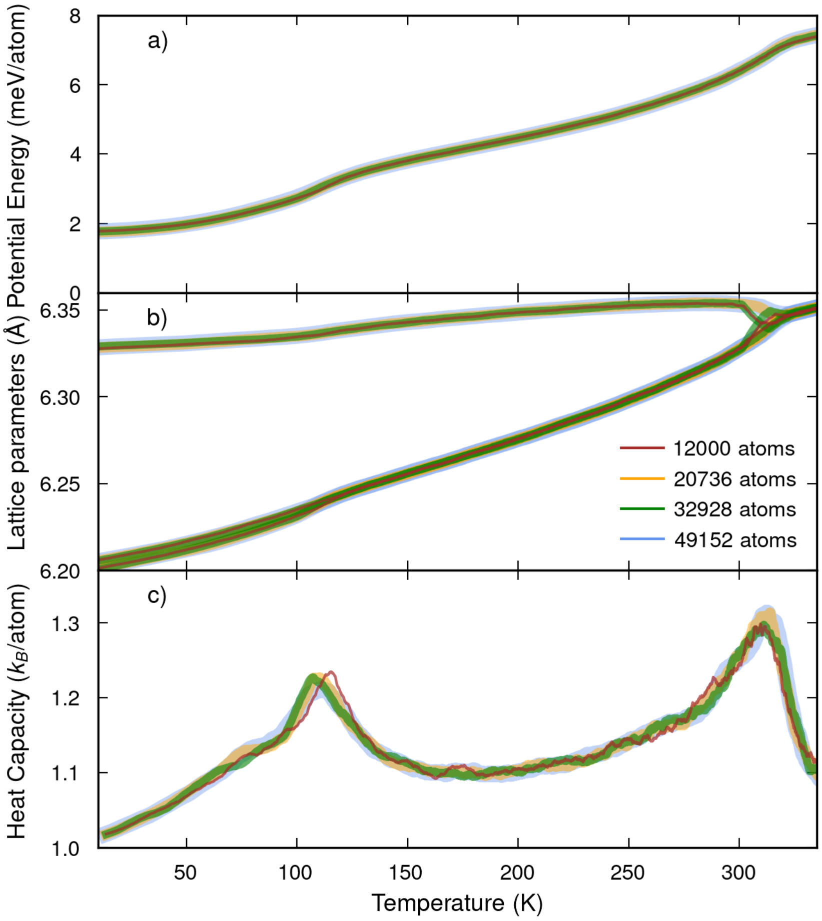


**Fig. S4:** (a) Potential energy, (b) lattice parameters, and (c) heat capacity, respectively, from heating and cooling molecular dynamics (MD) with 6.34 K/ns rate, for different system sizes in $FAPbI_3$. The different widths of the lines are used for better visibility.

- **Evolution of unit-cell volume from a cooling MD run: comparison between NEP and literature**

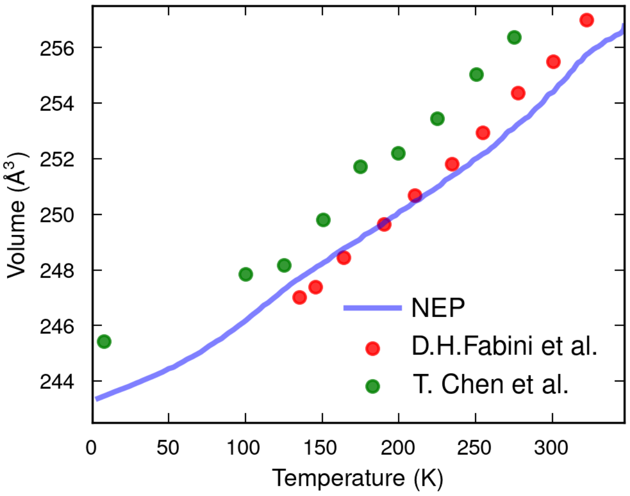


**Fig. S5:** The unit-cell volume per formula unit as a function of temperature during cooling MD simulation in $FAPbI_3$.The green and red symbols represent the data obtained from previous experiments for comparison [1, 2].

- **Evolution of angles betweeen three cell vectors from cooling and heating MD run**

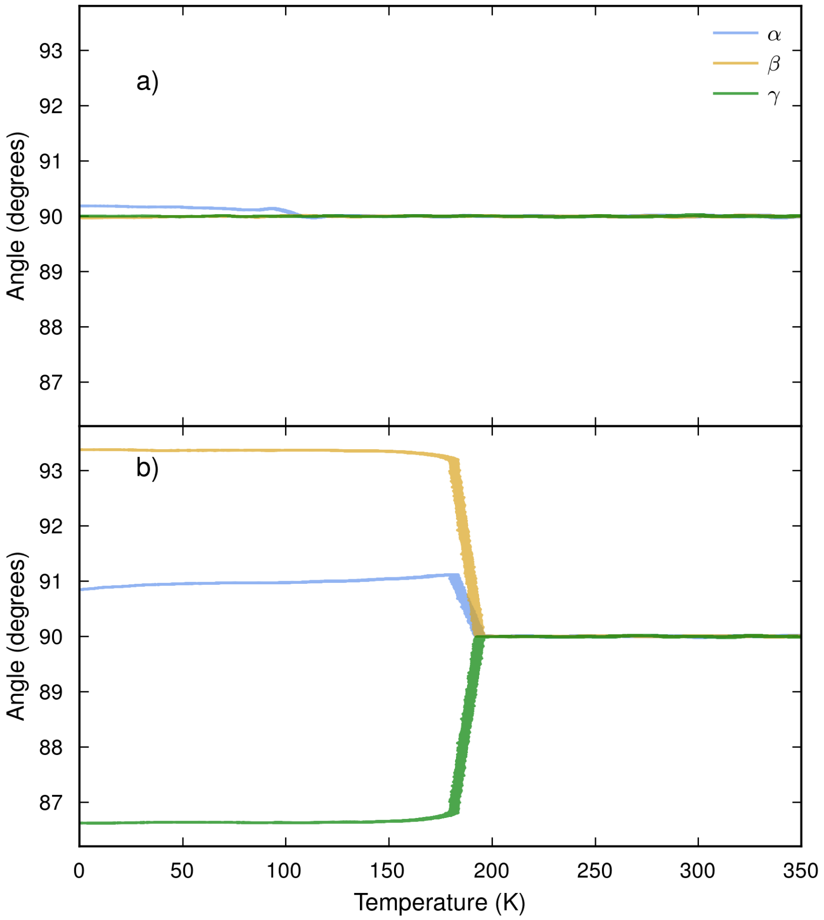


**Fig. S6:** Angles $\alpha$, $\beta$, and $\gamma$ between the three cell vectors of the 49152-atom supercell as a function of temperature in $FAPbI_3$ during the MD runs for (a) cooling and (b) heating.

- **Schematic representation of octahedral tilting and FA molecule, highlighting the two rotational axes**

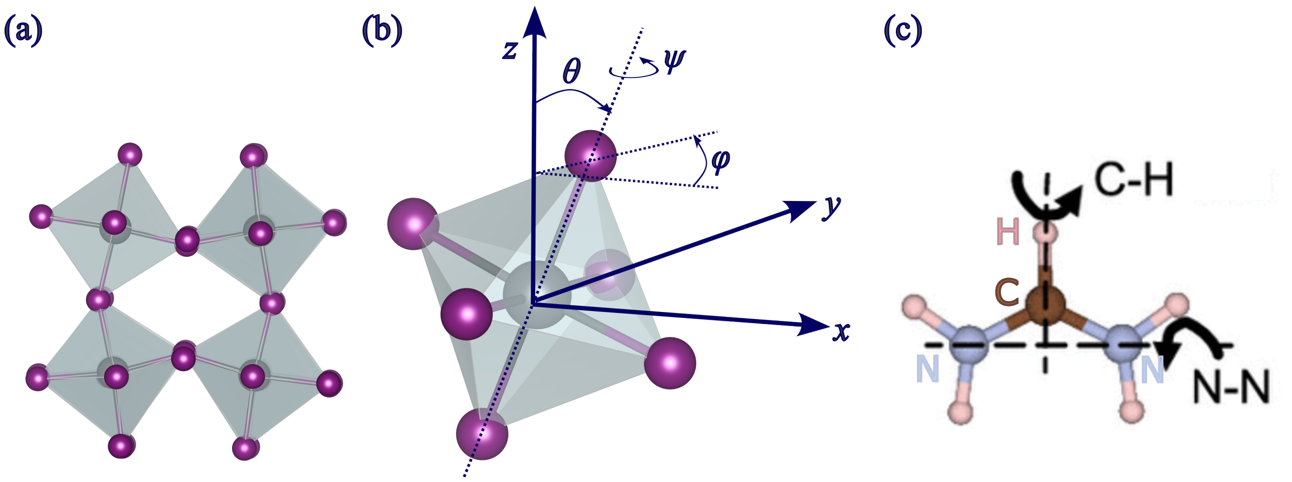


**Fig. S7:** (a) $FAPbI_3$ is represented using $PbI_6$ octahedra. (b) define the three Euler angles $\theta$, $\phi$, and $\psi$ and describe the octahedron's orientation. (c) represent the FA molecule indicating two rotational axis N−N, and C−H, reproduced from Ref. 3.

- **Snapshots of different phases from the cooling MD run**

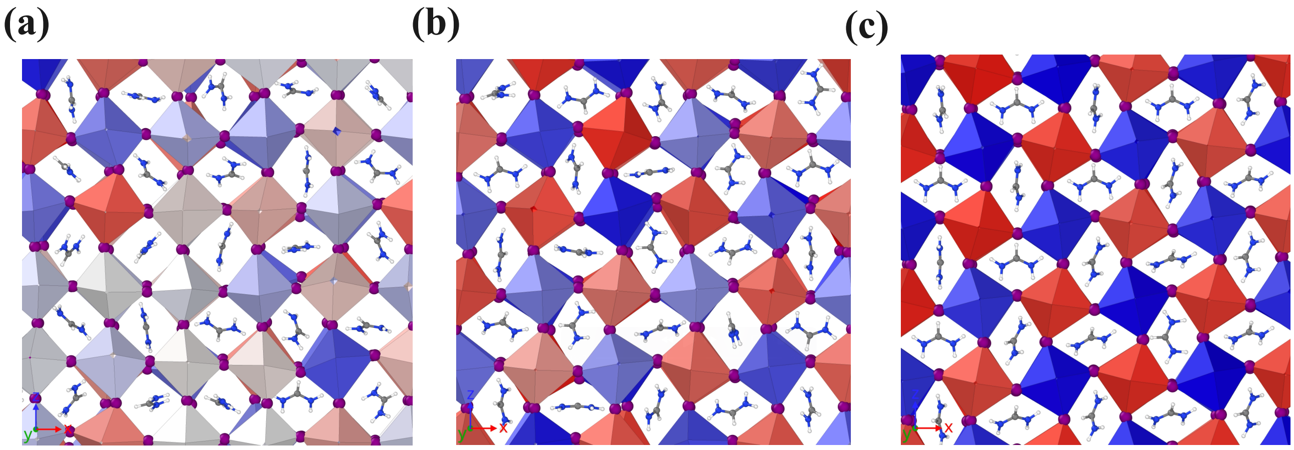


**Fig. S8:** Snapshots from the cooling MD run, illustrating the evolution of octahedral tilts in $FAPbI_3$ at different temperatures (a) 330 K ($a^0a^0a^0$), (b) 220 K ($a^0a^0c^+$), and (c) 10 K ($a^-a^-c^+$). The color coding of the octahedra correspond to tilt angle along the z-axis (out of the screen/paper), where red corresponds to -15° and blue to 15°.

- **Free energy landscape of the N–N vector of FA molecule at 200 K from the cooling MD run**

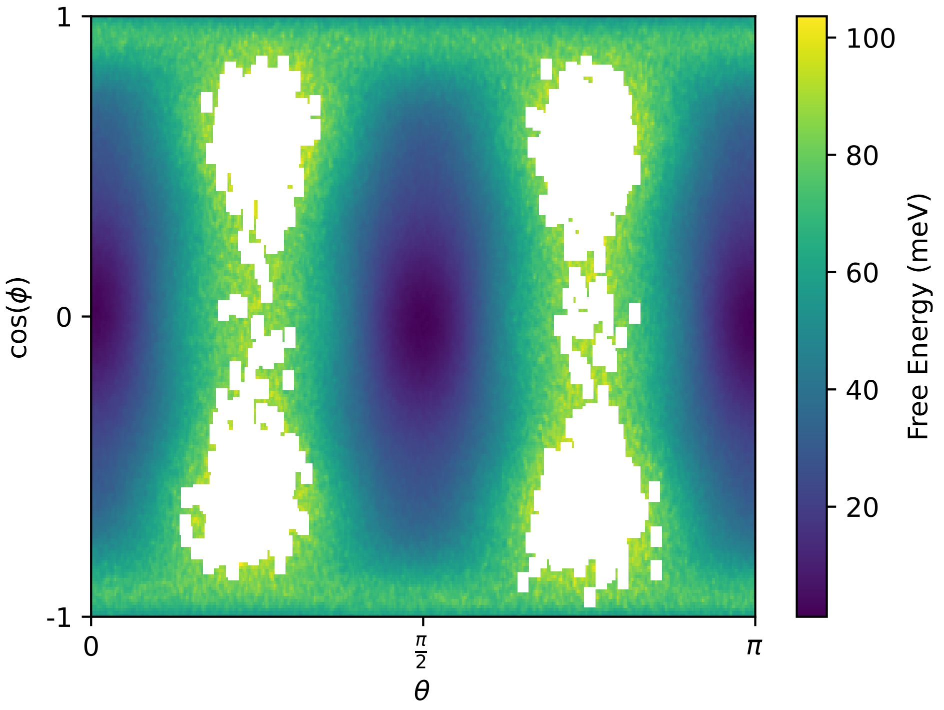


**Fig. S9:** Free energy distribution $F(\theta, \phi)$ of N–N vectors at 200 K in $a^0a^0c^+$-phase of $FAPbI_3$. Here, $\theta$ refers to angle in the $x-y$ plane and $\phi$ is angle to the $z$-axis. The white regions correspond to undefined free energy where the probability density of the N–N vectors is zero.

- **Nearest neighbour correlation analysis of N–N and C–H vector of FA molecule from cooling MD run**

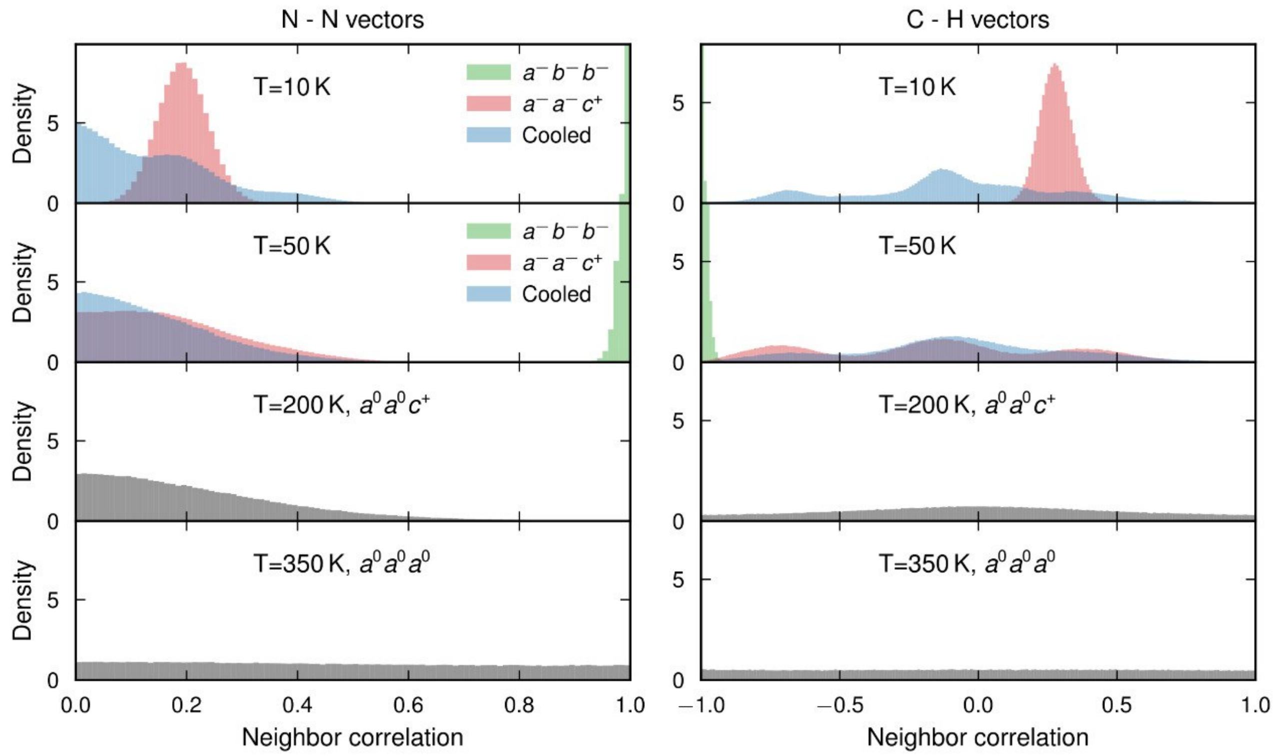


**Fig. S10:** Nearest neighbor correlation of N−N ($r^i_{NN} \cdot r^j_{NN}$ ) and C−H ($r^i_{CH} \cdot r^j_{CH}$ ) vectors in $a^-b^-b^-$ (in green), ideal $a^-a^-c^+$ (in pink) and $a^-a^-c^+$ obtained from MD cooling run (in blue), where 0 indicates two vectors are orthogonal to each other, and 1 indicates they are perfectly aligned.

- **Auto correlation function (ACF) of the N–N vector for each FA molecule in the system at different temperatures**

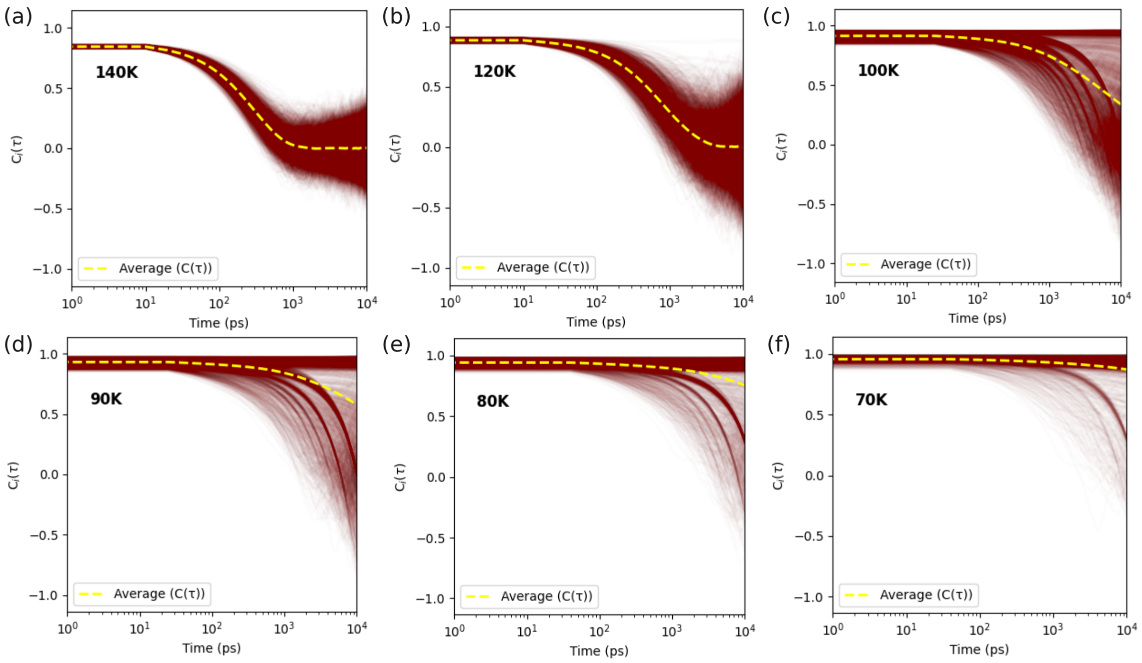


**Fig. S11:** The ACF of N–N for each FA molecule in the system as a function of time before and after second phase transition, at (a) 140 K, (b) 120 K, (c) 100 K, (d) 90 K, (e) 80 K, and (f) 70 K. The number of frozen FA molecules increases with decreasing the temperature.

- **Analysis of the ACF of the N–N and C–H vectors in the ground state (GS) phase**

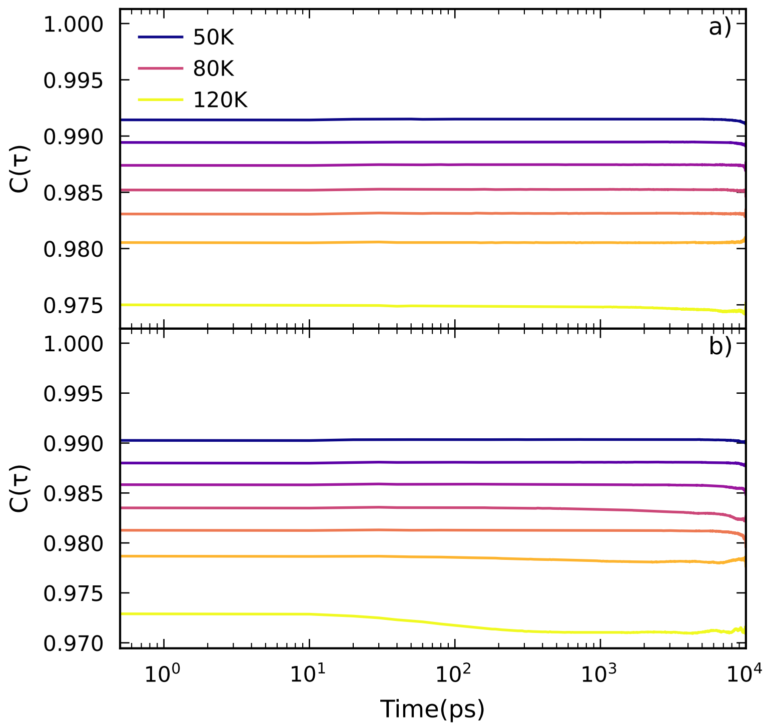


**Fig. S12:** Autocorrelation function $C(\tau)$ for the orientation of (a) N–N and (b) C–H vector in FA units in the GS phase ($a^-b^-b^-$). The spacing between the line is 10 K.

- ## Determination of NMR cooling rate

To estimate the cooling time of the sample, we considered that the sample was a cylinder (3.2 mm diameter, $d$; 15.4 mm height, $l$) made of sapphire, containing 30 mg of $FAPbI_3$ (mass, $m$) with a specific heat capacity ($c_p$) of $0.3\,\mathrm{J\,g^{-1}\,K^{-1}}$ (taking the value previously measured for the related $MAPbI_3$)[4]. Given the very high thermal conductivity of sapphire at 100 K ($2000\,\mathrm{W\,m^{-1}\,K^{-1}}$ to $4000\,\mathrm{W\,m^{-1}\,K^{-1}}$), we assume the rotor equilibrates with the environment almost instantaneously, and only the thermal mass of the powder limits the cooling rate. The sample was inserted into an environment at 95 K, with cooling provided by a high-flow (2000 L/min) of nitrogen gas at 95 K. The surface area exposed to the gas was calculated as

$$A = \pi d l + 2\pi \left(\frac{d}{2}\right)^2 = 1.71 \times 10^{-4}\,\mathrm{m^2}$$

Assuming a heat transfer coefficient of $h = 1000\,\mathrm{W\,m^{-2}\,K^{-1}}$, the cooling constant was determined as

$$k = \frac{hA}{mc_p} = \frac{1000 \times 1.71 \times 10^{-4}}{3.0 \times 10^{-5} \times 300} = 19.00\,\mathrm{s^{-1}}$$

Using Newton's law of cooling,

$$T(t) = T_{\mathrm{env}} + (T_{\mathrm{initial}} - T_{\mathrm{env}})e^{-kt}$$

the time to cool from $T_{\mathrm{initial}} = 298\,\mathrm{K}$ to $T_{\mathrm{env}} = 100\,\mathrm{K}$ was calculated to be approximately 0.2 seconds. This corresponds to a cooling rate of approximately $-3900\,\mathrm{K\,s^{-1}}$. This result is consistent with empirical experience in the Magic angle Spinning-Dynamic Nuclear Polarization (MAS DNP) community, where solvents such as toluene (melting point 178 K) are observed to freeze within less than one second after inserting into the LTMAS probe at 95 K.

# Supplementary Tables

- ## Total Energies of Low-Lying FAPbI Structures from DFT and NEP

**Table S1:** Total energies (in meV/atom) of the low-lying $FAPbI_3$ structures identified from our structural search, calculated using DFT and NEP potentials.

| Structures | DFT | | NEP | |
|---|---|---|---|---|
| | Energy | Reference Energy | Energy | Reference Energy |
| $a^-b^-b^-$ | -19.6431 | 0.0 | -19.6451 | 0.0 |
| $a^0b^-b^-$ | -19.6417 | 1.7 | -19.6445 | 0.6 |
| $a^0a^0c^+$ | -19.6394 | 3.7 | -19.6427 | 2.4 |
| $a^-a^-c^+$ | -19.6391 | 4.0 | -19.6435 | 1.6 |

- **Reference Energies of FAPbI Structures Relative to the abb Ground State, Calculated with Different ExchangeCorrelation Functionals Using DFT and NEP**

| XC-Functional | Negative Tilt | | | Positive/Mixed Tilt | | |
|---|---|---|---|---|---|---|
| | $a^-b^-b^-$ | $a^0b^-b^-$ | $a^-a^-a^-$ | $a^-a^-c^+$ | $a^0a^0c^+$ | $a^+a^+a^+$ |
| LDA | **0.0** | 0.0 | 0.6 | 0.6 | 1.2 | 2.5 |
| PBE | 0.0 | **-0.4** | 0.6 | 0.15 | 2.4 | 1.9 |
| PBE-sol | 0.0 | **-0.4** | -0.2 | 1.2 | 1.0 | 0.9 |
| PBE0 | 0.0 | **-1.4** | 3.7 | -0.9 | 1.4 | 4.8 |
| SCAN | 0.0 | **-0.17** | 0.64 | 2.12 | 3.0 | 1.0 |
| SCAN-vv10 | **0.0** | 1.4 | 3.1 | 4.0 | 3.7 | 1.42 |
| SCAN0 | 0.0 | **-0.15** | 0.7 | 2.2 | 3.0 | 1.02 |
| SCAN0-vv10 | **0.0** | 0.1 | 0.9 | 1.0 | 1.7 | 1.1 |

**Table S2:** Reference energy (meV/atom) of each structure relative to the DFT and NEP predicted ground state structure $a^-b^-b^-$ (using SCAN-vv10) for various XC functionals under different tilt conditions in meV/atom.

- **Experimental parameters for reported solid-state NMR measurements**

**Table S3:** Summary of experimental parameters for all solid-state NMR measurements reported in this work.

| Sample | Experiment | Recycle Delay (s) | Number of Scans | Experiment Time (minutes) |
|---|---|---|---|---|
| freeze 1 | $\{^1H\}$-$^1H$-$^{13}C$ CP | 2.0 | 624 | 21 |
| | $\{^1H\}$-$^1H$-$^{15}N$ CP | 2.0 | 3400 | 116 |
| freeze 2 | $\{^1H\}$-$^1H$-$^{13}C$ CP | 2.0 | 624 | 21 |
| | $\{^1H\}$-$^1H$-$^{15}N$ CP | 2.0 | 3908 | 133 |
| freeze 3 | $\{^1H\}$-$^1H$-$^{13}C$ CP | 2.0 | 624 | 21 |
| | $\{^1H\}$-$^1H$-$^{15}N$ CP | 2.0 | 2068 | 70 |

# Supplementary References


[1] T. Chen, W.-L. Chen, B. J. Foley, J. Lee, J. P. C. Ruff, J. Y. P. Ko, C. M. Brown, L. W. Harriger, D. Zhang, C. Park, M. Yoon, Y.-M. Chang, J. J. Choi, and S.-H. Lee, Proceedings of the National Academy of Sciences **114**, 7519 (2017).

[2] D. H. Fabini, C. C. Stoumpos, G. Laurita, A. Kaltzoglou, A. G. Kontos, P. Falaras, M. G. Kanatzidis, and R. Seshadri, Angewandte Chemie International Edition **55**, 15392 (2016).

[3] R. Lavén, M. M. Koza, L. Malavasi, A. Perrichon, M. Appel, and M. Karlsson, The Journal of Physical Chemistry Letters **14**, 2784 (2023).

[4] N. Onoda-Yamamuro, T. Matsuo, and H. Suga, Journal of Physics and Chemistry of Solids **51**, 1383 (1990).
